# A Case of Dronedarone-Induced Hypotensive Shock

**DOI:** 10.7759/cureus.8478

**Published:** 2020-06-06

**Authors:** Mohab Hassib, Ahmed Elkhouly, Shahryar Ansari, Steven Hamilton, Sowjanya Kapaganti

**Affiliations:** 1 Internal Medicine, St. Francis Medical Center, Trenton, USA

**Keywords:** dronedarone, atrial fibrillation, hypotensive shock, hypotension

## Abstract

Atrial fibrillation (AF) is the most common cardiac arrhythmia. Dronedarone is a new antiarrhythmic used for rhythm and rate control in AF and is associated with reduced mortality in non-permanent AF. It has also been associated with increased mortality in those with heart failure and/or permanent AF. This case report presents the case of hypotensive shock after initiating treatment with dronedarone in a 73-year-old female patient. Shortly after her first dose of dronedarone, she developed hypotension that did not respond to intravenous fluids and was admitted to the intensive care unit for vasopressor administration. Dronedarone was the most likely cause of the hypotension as the patient's blood pressure normalized in 24 hours, which concurs with the half-life of dronedarone. Amiodarone and dronedarone are similar in composition; however, the absence of iodine moieties in dronedarone contributes to its improved side-effect profile. Furthermore, amiodarone has been linked to hypotensive shock likely due to a co-solvent used in some intravenous preparations; however, dronedarone-induced hypotension is less common.

## Introduction

Atrial fibrillation (AF) is the most common sustained cardiac arrhythmia. AF treatment encompasses rate and rhythm control, thromboembolic prophylaxis, and treatment of the underlying disease, if any. Dronedarone, a non-iodinated analog of the class III antiarrhythmic, amiodarone, is a novel anti-arrhythmic drug that has proved to provide rhythm and rate control in non-permanent AF patients. It has been associated with reduced mortality and hospitalization in those with non-permanent AF and an increase in mortality in those with heart failure and/or permanent AF [1,2]. The objective of this study is to shed light on an unexpected case of severe hypotension leading to shock hours after beginning treatment with dronedarone in a patient with AF.

## Case presentation

**Figure 1:**
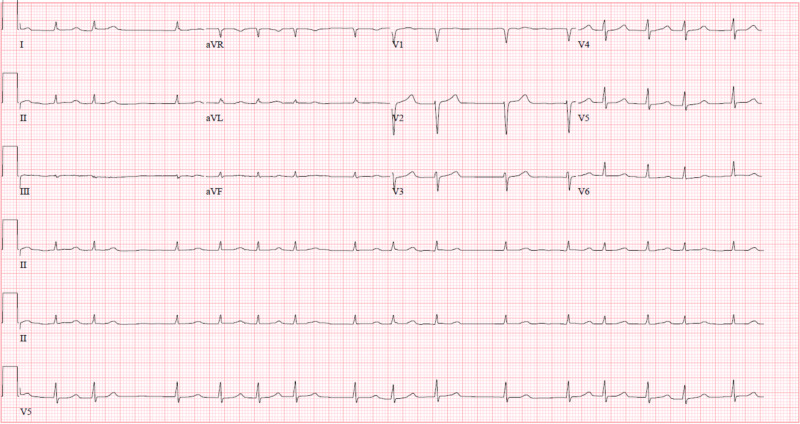
EKG on admission showing atrial fibrillation.

A 73-year-old female presented to the clinic and was found to have AF. She had a medical history of hypertension, type 1 diabetes mellitus, coronary artery disease, peripheral vascular disease, and hyperlipidemia. She was started on dronedarone at a dose of 400 mg orally twice daily. Her other cardiac medications were carvedilol, diltiazem, amlodipine, furosemide, and rivaroxaban. She took the first dose of dronedarone at 7 pm that evening. Twenty minutes later, she felt dizzy and lost consciousness and was admitted to the emergency department. She was found to be hypotensive at 76/53 mmHg on admission. Her remaining vitals on admission are as follows: temperature of 36.2°C, respiratory rate of 22 breaths per minute, heart rate of 96 bpm and irregularly irregular, and oxygen saturation of 100% on 3L/minute nasal cannula. Her EKG showed AF with a heart rate of 96 bpm and a QTc interval of 463 msec (Figure 1). Arterial blood gas analysis was as follows: pH of 7.36, PCO_2_ of 27.5, PO_2_ of 114.3, and HCO_3-_ of 15.0, displaying a borderline metabolic acidosis with a normal anion gap and normal lactate level. In the emergency department, she received 4 liters of 0.9% normal saline in intravenous boluses, with no improvement in her hypotensive episode whatsoever.

She was admitted to the intensive care unit for persistent hypotensive shock. Blood test results were unremarkable apart from hyperkalemia and elevated creatinine denoting an acute kidney injury secondary to hypotensive shock. Her respiratory rate worsened to 35 breaths per minute, and she was intubated and ventilated. Her EKG showed a prolonged QTc of 519 msec. She was then given intravenous vasopressors, norepinephrine, and phenylephrine, which improved the blood pressure. She spent a total of one day in the intensive care unit. The following day, her blood pressure improved as vasopressors were weaned and she was extubated and transferred to the general medical floor. No significant events were encountered on the medical floor and she was eventually discharged home three days later on a therapeutic dose of amiodarone.

## Discussion

Chemically, dronedarone is a benzofuran derivative related to amiodarone. The use of amiodarone, however, is limited by its side effects due to its high iodine content, which can lead to pulmonary fibrosis, thyroid disease, liver disease, and severe cases of hypotensive shock. These cases of hypotensive shock were attributed to a co-solvent used in intravenous amiodarone preparations, which lead to hypotension due to either anaphylactic reactions or severe left ventricular dysfunction, which is much rarer.

In dronedarone, the iodine moieties are not present, which reduces the toxic effects on the thyroid and other organs. In addition, a methylsulfonamide group is added to reduce solubility in fats (lipophobicity), therefore reducing neurotoxic effects [3]. Dronedarone displays amiodarone-like class III antiarrhythmic activity in vitro and in clinical trials [4,5]. The drug also appears to exhibit activity in each of the four Vaughan-Williams antiarrhythmic classes [5]. This activity might be the reason for severe hypotension developing in our patient after dronedarone use. This is evidenced by the prompt cessation of her hypotension corresponding with the approximate half-life of dronedarone, that is, 13-19 hours.

## Conclusions

We concluded that our patient’s hypotension was most probably due to an uncommon side effect of dronedarone. This is due to the fact that she had severe hypotension and shock shortly after taking her first dose of dronedarone, and the resolution of her hypotension was in parallel with the approximate half-life of the drug.
